# A New Method for Imputing Censored Values in Crossover Designs with Time-to-Event Outcomes Using Median Residual Life

**DOI:** 10.1155/2020/8475154

**Published:** 2020-12-10

**Authors:** Maryam Jalali, Zahra Bagheri, Najaf Zare, Seyyed Mohammad Taghi Ayatollahi

**Affiliations:** ^1^Department of Biostatistics, Medical Schools, Shiraz University of Medical Sciences, Shiraz, Iran; ^2^Department of Biostatistics, Infertility Research Center, Shiraz University of Medical Sciences, Shiraz, Iran

## Abstract

Crossover designs are commonly applied in research due to efficiency and subject parsimony compared to parallel studies. Baseline measurements would improve the power of comparison. For time to event outcomes, the sample size is reduced due to censorship, if they are ignored; thus, applying traditional regression models will be limited. A logical solution is to impute the censored observation and apply common analytical models for analyzing the data. Nevertheless, techniques to impute censored data in time-to-event outcomes in crossover designs are not practiced as much. Accordingly, we propose a method to impute the censored observation using median residual life regression and then analyze the data using analyses of covariance (ANCOVA), considering the difference of period-specific baselines as covariate. We used simulation to show the favorable performance of our method relative to a recently proposed method, multiple imputation with model averaging and ANCOVA (MI^MI^). Specifically, the censored observations were multiply-imputed using prespecified parametric event time models, and then, the methods were applied to a real data example.

## 1. Introductions

In clinical trial designs, to compare the effect of treatment on the same subject over different treatment periods, crossover designs are appropriate due to higher accuracy and subject parsimony [1]. Two-treatment two-period crossover is the simplest design with two treatments, A and B, where subjects are randomly allocated to AB or BA sequences [2].

Based on previous studies, consideration of the baseline measurement in the analysis of crossover design, which is gathered before receiving treatment, can increase the power of comparisons [3–5]. The literature suggests that the analysis of covariance (ANCOVA), considering within subject differences in baseline responses as a covariate, has a good performance in crossover design [3, 4, 6–9].

Time-to-event endpoints in crossover clinical designs are also commonly investigated. For example, in a crossover trial of pregabalin for neurogenic claudication, the outcome variable was “the time to first moderate pain symptoms during a 15-minute treadmill test.” Markman et al. studied the efficacy of an oral spray involving treadmill exercise testing [10, 11]. In both mentioned studies, baseline measurements were gathered, but not considered in the analysis. Xu et al. examined a crossover trial in a treadmill walking test. The recorded time to a specific cardiopulmonary event was the main outcome, and the participants who walked more than 10 min were considered right censored observation. Every participant had four responses: baseline and posttreatment responses for periods 1 and 2. They applied analysis of covariance (ANCOVA) to consider the baseline measurements in estimating the drug effect [1].

For ethical reasons, it is essential to keep the number of patients in a clinical trial as low as possible. As evidenced by extensive research publications, crossover design can be a useful and powerful tool to reduce the number of patients required for a parallel group design [12, 13]. An explicit statistical issue in regression modeling is that adequate statistical power cannot be reached in a small sample size (which is a characteristic of crossover designs) [14]. Apparently, in time-to-event endpoints, censorship is inevitable; thus, ignoring censored responses can lead to fewer sample sizes and critical conditions. The alternative is to impute the censored values as real as possible before data analysis.

Xu et al. incorporated multiple imputation (MI) of censored values and ANCOVA to consider baseline measurements in analyzing 2 × 2 crossover studies with censored time-to-event outcomes. The superiority of their method over the hierarchical rank test was demonstrated through simulations [1, 15]. Their imputation method was based on fitting parametric models and using MI. Finally, they applied Rubin's rule to estimate the treatment effect. In spite of satisfactory results, the computation and imputation process was cumbersome and time-consuming [1].

Although matching is done in crossover design, there are some variables that can affect the duration of a time-to-event outcome before receiving any treatment. For example, regardless of the study type, in a treadmill test, age is an effective factor on the walking duration. Hence, it would be beneficial to apply a method that incorporates demographic variables in the imputation step. In addition, imputation would be more precise, if we consider the censored times and use them as a predictive input when imputing them. However, these were not included in the aforementioned method.

The use of advanced statistical indices would improve statistical analysis. The mean residual life function and the quantile (median) residual life function are functional indices that are usually applied to summarize the survival experience of patients [16]. The mean residual life function has some limitations, though. Firstly, it cannot be estimated reliably in the presence of censored observation and secondly; as it extremely depends on outliers, it is very unstable even in completed data [16].

In this paper, we develop a new approach that can be used directly to infer the effect of covariates on the median residual lifetime (MERL) at any specific time point, to predict actual lifetimes more accurately.

After imputing the predicted censored observations in crossover time-to-event data based on MERL regression models, then, we apply ANCOVA to consider the baseline measurements and treatment effect. Specifically, two models will be discussed: (i) the imputation model which is the main aim of this study which is applied for imputing censored values and (ii) the analytical model, ANCOVA, which is performed similar to the Xu et al. study [1].

To impute the censored observations in the first part, we applied the median residual life regression model, which has not been used for this purpose in crossover designs and is free from the mentioned limitations.

Section 2 presents details of the proposed method. In Section 3, we contrast the numerical performance of our proposed method with the multiple imputation method through simulation studies.  Section 4 presents the results from applying the different methods to a real data example.  Section 5 deals with discussion.

## 2. Methods

We assumed a 2 × 2 crossover trial with two treatments (A and B) and two sequences (AB or BA) with a wash-out period between periods 1 and 2. The baseline and posttreatment responses are indicated as *b*_*ijk*_ and *Y*_*ijk*_, where *i* = 1, 2 represents periods; *j* = 1, 2, ⋯, *n* denotes subjects; and *k* = 1, 2 shows sequences. It can be assumed that after logarithm transformation, (*b*_1*j*1_, *Y*_1*j*1_, *b*_2*j*1_, *Y*_2*j*1_)^*T*^ and (*b*_1*j*2_, *Y*_1*j*2_, *b*_2*j*2_, *Y*_2*j*2_)^*T*^ follow a multivariate distribution with different means and the same variance-covariance structure *Σ*. If a subject dose not experience the event during the follow-up time, he will be assumed as a censored observation at the end of period time which is shown by time *τ*. Another assumption is that there is no censoring observation at baseline.

In our method, first, the censored observations are imputed by applying the predicted median residual life regression added to the censored time, which is the main objective of our study. Then, similar to Xu et al.'s study, ANCOVA is done to estimate “the ratio of geometric means of the event times for treatment relative to B,” which is represented by *θ*.

Next, the null hypothesis H0∶*θ* = 1 is tested. For symmetric distributions (on the log scale), the geometric mean is equivalent to the median. Thus, this parameter can be used for estimating the ratio of median survival of the two treatments, which is usually of interest in survival analysis.

### 2.1. Imputation Model

#### 2.1.1. Imputing Censors by Median Residual Life Regression

The median residual life function is as follows:

(1) $$\begin{array}{c} m\left(t_{0}\right)=\text{median}\left(\text{Ti}-t_{0}\;|\;\text{Ti}>t_{0}\right), \end{array}$$

This is interpreted as “the remaining lifetimes among survivors beyond time *t*_0_”.

To adjust for confounding variables and consider them as covariates, regression modeling is a proper choice.

Suppose that *z*_1*i*_ is a bivariate covariate that 0 and 1 are assigned to the control and treatment groups, respectively. Applying the invariance property of the median, the median residual life repression equation is written as follows [16]:

(2) $$\begin{array}{c} \text{Median}\left(\text{Ti}-t_{0}\left|\text{Ti}>t_{0},z_{1i}\right.\right)=\exp\left(\beta_{t_{0}}^{\left(0\right)}+\beta_{t_{0}}^{\left(1\right)}z_{1i}\right). \end{array}$$

Now, in our study, we impute the censored times using median residual life regression. First, this is done for the censored observations in period 1 and afterwards, censored times in period 2.

We fit the above model and use baseline information in period 1, age and treatment indicators as covariates, and then compute the median residual life regression for every censored observation:

(3) $$\begin{array}{c} \text{Median }\left(\text{Ti}-t_{0}\;|\;\text{Ti}>t_{0},z_{1jk},{\text{age}}_{\begin{array}{c} 1jk\\ \; \end{array}},b_{1jk}\right)=\exp\;\left(\beta_{t_{0}}^{\left(0\right)}+\beta_{t_{0}}^{\left(1\right)}z_{1i}k+\beta_{t_{0}}^{\left(2\right)}A_{1ik}+\beta_{t_{0}}^{\left(3\right)}b_{1ik}\right),\;j=1,2,\cdots ,n,k=1,2 \end{array}$$

where *z*_1*jk*_ is the treatment indicator, *A*_1*ik*_ represents the age of each subject in period 1, and *b*_1*ik*_ denotes the baseline measurement of each subject. The corresponding complete set of uncensored post treatment values in period 1 is denoted by *Y*_1*jk*._

After completing the censored time in period 1, the censored values in period 2 are imputed by the regression equation ahead:

(4) $$\begin{array}{c} \text{Median }\left(\text{Ti}-t_{0}\;|\;\text{Ti}>t_{0},z_{2jk},{\text{age}}_{\begin{array}{c} 2jk\\ \; \end{array}},b_{2jk},Y_{1jk}\right)=\exp\;\left(\beta_{t_{0}}^{\left(0\right)}+\beta_{t_{0}}^{\left(1\right)}z_{2ik}+\beta_{t_{0}}^{\left(2\right)}A_{2ik}+\beta_{t_{0}}^{\left(3\right)}b_{2ik}+\beta_{t_{0}}^{\left(3\right)}b_{1ik}+\beta_{t_{0}}^{\left(4\right)}Y_{1jk}\right). \end{array}$$

In addition to the mentioned variables, *Y*_1*jk*_ also is added to the equation. The corresponding complete set of uncensored post treatment values in period 2 is represented by *Y*_2*jk*_.

For each censored value, *t*_0_ is set to the censorship time itself as the idea is to find the residual life time after *t*_0_. After computing the median residual time for every censored time, the final imputed time is obtained through summing the median residual life time and the censored time for that person.

### 2.2. Analysis of Covariance

After imputing censored times, ANCOVA is applied to the completed data on the log scale. The difference between posttreatment event times *∆*_*jk*_ = log(*Y*_1*jk*_) − log(*Y*_2*jk*_) is regressed on the difference between baseline values *D*_*jk*_ = log(*b*_1*jk*_) − log(*b*_2*jk*_) and the sequence indicator *Q*_*j*_:

(5) $$\begin{array}{c} {∆}_{jk}=\gamma_{0}+\gamma_{1}\;D_{jk}+\gamma_{2}\;Q_{j}+\varepsilon_{jk},\;\varepsilon_{jk}∼N\;\left(0,\eta^{2}\right). \end{array}$$

The point estimator for the geometric mean for treatment A relative to B is log$\hat{\theta }=\hat{\gamma_{2}}/2$.

The *P* value for the hypothesis H0∶*θ* = 1 is obtained from the *P* value of the test $\text{H}0∶\log\hat{\gamma_{2}}=0$ in the ANCOVA output.

## 3. Simulation

### 3.1. Simulation Set-Up

We carried out a simulation study to compare the performance of our proposed and multiple imputation method. Type 1 error and power are reported for both methods. The bias and 95% confidence interval coverage probability for *θ* are reported too.

The distributions considered for event time in the simulations were *Exponential*, *Weibull*, and *Gamma*, which are commonly applied for survival data.

Multivariate correlated event times from *Exponential* were generated with mean (.3, .3*θ*, .3, .3)^*T*^ for AB sequence and (.3, .3, .3, .3*θ*)^*T*^ for BA sequence with common variance-covariance structure and correlation coefficients.

*Weibull* distribution with shape parameter *s* and decay parameter *d* has the density of

(6) $$\begin{array}{c} \begin{array}{c} f\left(x\right)=s\times d\times x^{\left(s-1\right)}\times \exp\left(-d\times x^{s}\right),\\ \text{For }x\geq 0;s,d>0. \end{array} \end{array}$$

For the Weibull distribution, the shape parameter was (0.95,0.95,0.95,0.95)^*T*^ for both sequences and the decay parameter was (0.29,0.29*θ*, 0.29,0.29)^*T*^ for AB sequence and (0.29,0.29,0.29,0.29*θ*)^*T*^ for BA sequence [17].

The third distribution, *Gamma*, was generated for better presenting the performance of our method. The correlated event time for Gamma distribution was generated with the same shape (3.2, .3.2,3.2,3.2)^*T*^ for both sequences and rate (.7, .7*θ*, .7, .7)^*T*^ for AB sequence and (.7, .7, .7, .7*θ*)^*T*^ for BA sequence with common variance-covariance structure and correlation coefficients [17].

The parameters for generating multivariate distributions were selected such that the generated data would be approximately similar to the motivating data example in Xu et al.'s study. They were selected such that the data would fall within the range of 0-10 with a mean of about 5 for each period [1].

We considered the first-order autoregressive (AR(1)) for the correlation structure as it is usually similar to the real data correlation structure. The AR(1) correlation structure is as follows:

(7) $$\begin{array}{c} \left(\begin{array}{cc} \begin{array}{cc} 1 & \rho \\ \rho & 1 \end{array} & \begin{array}{cc} \rho^{2} & \rho^{3}\\ \rho & \rho^{2} \end{array}\\ \begin{array}{cc} \rho^{2} & \rho \\ \rho^{3} & \rho^{2} \end{array} & \begin{array}{cc} 1 & \rho \\ \rho & 1 \end{array} \end{array}\right). \end{array}$$

The mean correlation coefficient $\bar{\rho }$ was considered 0.5 and 0.7: *ρ* = 0.7 for $\bar{\rho }=0.5$ and *ρ* = 0.83 for $\bar{\rho }=0.7$. These are the same as Xu et al.'s study. These means of correlation are used to keep a reasonable level of intersubject correlation in the generated data.

We assumed that censorship did not occur at baseline time and the case of censorship was right-censoring in the posttreatment time at *τ*. The parameter of study, *θ*, is the ratio of the geometric means of the event times for treatment A and treatment B.

The performance of different models was compared in distinct scenarios of sample size, correlation coefficients, and percentage of censoring.

The sample size per sequence was considered *N* = 12, 24, 48, and the percentage of censoring was 30% and 50% for the total sample. Each scenario was repeated 1000 times: *θ* = 1, under the null hypothesis. Under the alternative hypothesis, the value of *θ* was chosen such that the power was about 80% for the multiple imputation method proposed by Xu et al. in different scenarios of simulations [1]. The true *θ* values used in the simulation are shown in Table 1. All analyses and data generation were done in R software. lcmix package was applied for generating correlated multivariate datasets. In this package, the construction of multivariate distributions from univariate marginal distributions using normal copulas is discussed.

### 3.2. Simulation Results

Type 1 error for the proposed and multiple imputation method is shown in Table 2. The errors in all scenarios for the two methods were less than 5% and well controlled. Obviously, the Weibull distribution controlled the error more than Exponential.

For the Gamma distribution, the type one errors had a noticeable reduction in comparison to the Exponential and Weibull distribution, especially in 30% censoring.

Powers of the mentioned methods are shown in Table 2. In all distribution, powers of the proposed method were more or equal to the multiple imputation method. On average, the power of our proposed method was 11% more than the multiple imputation method in each mean pairwise correlation, irrespective of sample size, percentage of censoring, and distribution. When censoring was 30%, the average power of our method was 12% more than multiple imputation, irrespective of sample size, distribution, and mean pairwise correlation. This figure was 14% and 12% for the Gamma and Weibull distribution, respectively, irrespective of other scenarios of simulation.

For our proposed method, in each percentage of censoring, the power increased with increase in the sample size. The power for the Gamma distribution was greater than that of the other two distributions. Further, the power was higher when the mean pairwise correlation was 0.7 in comparison to 0.5 and the percentage of censoring was 30% in comparison to 50%.

The percentage of bias under the null and alternative hypothesis and 95% confidence interval (C.I.) coverage probability for log (*θ*) under the alternative hypothesis are reported in Table 3. The bias was not larger than 10% in all scenarios and was ignorable in all scenarios.

Further, the 95% C.I. coverage probability was kept at or more than the nominal level in every scenario.

## 4. Data Application

The type of data in this study is rare; hence, finding a suitable data that fulfills the condition of crossover design (time to event outcome) is very limited. Thus, we applied the data set from Xu et al.'s study [1]. This dataset is a 2 × 2 crossover clinical trial, where the effect of a drug was investigated in a treadmill test as discussed in the introduction. The data and its details are presented in Table 4.

The sample size was 40 in total, of which 20 were randomly allocated to the placebo-drug sequence and the other 20 to drug-placebo sequence, which was a treadmill walking test. Also, the time until an event related to cardio was considered the outcome. The follow-up time was 10 min. The baseline measurements were performed for each participant before receiving any treatment in both periods. The raw data have been presented in Xu et al.'s article [1]. After imputing the censored data by our proposed method and applying ANCOVA, there was a significant difference between the drug and placebo group (*P* value = 0.004). Our results were in line with the multiple imputation method (*P* value = 0.005). In addition, the ratio of geometric mean of time to the mentioned event (*θ*) was 1.78 (SE = 0.16), with 95% C.I. of (1.47, 2.09). This ratio was 1.67 (SE = 0.25), with 95% C.I. of (1.18, 2.35) in Xu et al.'s study [1].

Table 5 outlines the estimated survival time and standard errors (SE) of the censored observations in the treadmill test for the multiple imputation method and the proposed method with and without adding age as a covariate. The estimates for our method are more accurate than the multiple imputation method. Also, adding age to the model led to enhanced accuracy.

## 5. Discussion

To our knowledge, methods for imputing censored observations in time-to-event outcomes in small sample size data have been seldom discussed in the literature. One discussed method for imputing censored observation in time-to-event outcomes is restricted mean survival time. For example, Liu et al. as well as Grover and Gupta proposed a method based on multiple imputations to impute censored outcomes, using restricted mean survival time in small sample size data. Their method was similar, but the superiority of the second one was that the upper limit for imputation of censored survival time was taken as the highest survival time within the study, where Liu et al. set the upper limit as some reasonable value [18, 19]. Based on Liu et al.'s simulations, their method was much better than its competitor in respect of bias and efficiency. Survival estimates of restricted lifetime model parameters and marginal survival estimates had better precision.

Based on Grover's method, the estimates were better and the standard errors were smaller in comparison to the analysis of the data having censored observations in outcome but ignoring cases with missing covariates and censored outcomes. In both mentioned studies, the final achievements were similar to ours in terms of reducing bias and increasing precision, but there are some weakness, although statistical inferences based on mean survival time are appealing, but as discussed before, it has some limitations. Another aspect that should be noted is that if the observed survival time is too long or the follow-up time is very short, the mean survival time is not a good choice [20]. Multiple imputation leads to more complicated computation and work. Obviously in regression modeling, using covariates that are correlated to the response variable leads to the improvement of predictions [18]. Although, in these studies, demographic information was imbedded in the model of imputation, the superiority of our new method has been use of median residual life time instead of mean residual life time which is more robust. [16]. Additionally, the other advantage of our proposed method has been applying the information of censoring time, which was ignored in the mentioned studies.

We proposed a new method to impute censored observation in time-to-event outcomes in crossover designs. To our knowledge, this is the first study in which the median residual life regression has been applied to impute censored survival data based on correlated covariates. Our proposed method, median residual life regression, is free of the stated limitations. In addition to resolving the mentioned limitations, our method has two additional advantages. It considers the effect of censored time when imputing the life times for censored observations, by adding the time of censoring to the predicted median residual life, as well as its simplicity.

We showed that our method generated more efficient results in comparison to the recently presented method “multiple imputation” by Xu et al. [1], through different combinations of sample size, percentage of censoring, distribution, and mean pairwise correlation coefficient.

Based on our simulations, we found that our method had equal or higher power than the multiple imputation method proposed by Xu et al. In addition, there were no inflated type 1 errors and the entire bias was negligible under the null and alternative hypothesis. This is a notable achievement of our study. The reason is that Xu et al.'s method is based on considerable computation and multiple imputations. Furthermore, they applied complicated rules to combine the datasets in imputation steps, while our proposed method is straightforward and not that time-consuming. In addition, we pointed out some practical issues, which were ignored in the mentioned study. First of all, consideration of the confounding and effective factors in computing the median residual life might lead to a more accurate prediction of the censored observations. As there are many effective factors, a penalized method [21] can be applied to select the most important variables. Another advantage of our method is that the imputed time is added to the censored time, but the role of censoring time is not considered in the multiple imputation method.

The comparison of our study results with the results of multiple imputation method [1] showed that the results of both methods were almost the same and the *P* value of both methods when testing the effect of treatment was significant. The estimation of the parameters for both methods was close to each other, but the estimates from our method had less standard error and were more accurate. The comparison of imputed times for censored observations revealed that the imputation method based on our proposed model which used the age variable in imputation was more accurate than the multiple imputation model. It was even more accurate than our proposed method regardless of age and led to more accurate predictions.

Crossover designs can be performed in small sample sizes, such as 4 or 6. One limitation in our method is that it cannot be converged in the mentioned sample sizes, similar to Xu et al.'s method. Additionally, we considered 30% and 50% of censoring, while in real data a higher censoring percentage can be encountered. Another issue that should be pointed out is that censoring was independent of covariates in our study. Further studies are warranted to evaluate the performance of the discussed methods at higher percentages of censoring, for censoring which is not independent of covariates and for evaluating median residual life regression in imputing censors in all survival studies besides crossover designs.

## 6. Conclusion

Our proposed method outperformed the multiple imputation method. All the powers were greater than 80%, and there was no bias in estimating the parameter of interest. In addition, all type 1 errors were less than 5%. Thus, it is suggested for imputing censored observations given its simplicity and not very heavy computational work as well as its efficiency.

## Figures and Tables

**Table 1:** True *θ* values used in simulation study under the alternative hypothesis for each combination of distribution, mean pairwise correlation coefficient, percentage of censoring, and sample size.

Mean pairwise correlation	$\bar{\rho }=0.5$						$\bar{\rho }=0.7$					
Percentage of censoring	30%			50%			30%			50%		
*N*	12	24	48	12	24	48	12	24	48	12	24	48
Dist												
Exp	2.33	1.59	1.6	2.3	1.8	1.5	2.1	1.7	1.5	2	1.52	1.35
Weibull	2.3	1.93	1.68	2.3	1.69	1.51	2	1.78	1.56	2	1.64	1.28
Gamma	2.1	1.4	1.3	1.95	1.65	1.2	2	1.4	1.24	2.1	1.3	1.14

**Table 2:** Type 1 error (target is 5%) and power (%) for the multiple imputation with model averaging and analysis of covariance (*MI*^*MA*^) and our proposed method (1000 simulations).

Mean pairwise correlation			$\bar{\rho }=0.5$						$\bar{\rho }=0.7$					
Censoring percentage			30%			50%			30%			50%		
Distribution		*N*/seq	12	24	48	12	24	48	12	24	48	12	24	48
		Method												
Exp	Error	*MI* ^*MA*^	4.8	4	4.9	5	4.5	5	4.5	4.6	4.9	4.8	5	5
		MERL	4.6	4	4.8	4.9	4.1	5	3.9	4	4.4	3.8	4.1	4.5
	Power	*MI* ^*MA*^	78	80	81	69	79	80	81	79	84	78	80	81
		MERL	81	87	91	78	80	83	82	94	85	84	86	87
Weibull	Error	*MI* ^*MA*^	3.8	4	4.3	4	5	4	4.1	3	4.9	4.2	3.4	4.8
		MERL	3.5	4.1	4	4.2	4	4.3	3.8	3.1	4	4	3.5	4.2
	Power	*MI* ^*MA*^	79	80	81	72	80	81	79	80	78	81	79	80
		MERL	85	90	91	75	81	90	89	95	96	89	90	91
Gamma	Error	*MI* ^*MA*^	1.3	1.5	2.7	4.5	3.1	4.6	2.2	2.9	3.4	1.8	2.4	4.1
		MERL	1	1.2	2.1	4.1	4.1	5	1.9	2	3.4	2	2.1	3.9
	Power	*MI* ^*MA*^	77	80	79	75	78	80	81	81.9	79	81	83	82
		MERL	88	95	93	80	90	95	90	95	96	88	92	94

**Table 3:** Percentage of bias (%) under the null and alternative hypothesis and 95% confidence interval coverage probability under the alternative hypothesis *H*_1_:*θ* ≠ 1 for estimating log*θ* for our proposed method when the covariance structure is AR (1) and the distributions are exponential (E), Weibull (W), and Gamma (G).

Mean pairwise correlation		$\bar{\rho }=0.5$						$\bar{\rho }=0.7$					
Percentage of censoring		30%			50%			30%			50%		
Dist	*N/seq*	12	24	48	12	24	48	12	24	48	12	24	48

E	Bias (*H*_0_)	−3 × 10^−5^	−4 × 10^−5^	3 × 10^−5^	−2 × 10^−5^	−3 × 10^−5^	−3 × 10^−5^	45 × 10^−5^	−5 × 10^−5^	71 × 10^−5^	2 × 10^−5^	2 × 10^−5^	−3 × 10^−5^
	Bias (*H*_1_)	0.80	1	0.6	3	3.1	2.9	1.6	1.7	1.2	3	5	4.9
	*Coverage*	94.5	94.9	95	97	95	94.4	95.3	95	96.5	96	95.5	97

W	Bias (*H*_0_)	4 × 10^−5^	3 × 10^−5^	68 × 10^−6^	−1 × 10^−5^	−2 × 10^−5^	3 × 10^−5^	4 × 10^−5^	−3 × 10^−5^	5 × 10^−5^	5 × 10^−5^	2 × 10^−5^	−2 × 10^−5^
	Bias (*H*_1_)	0.6	0.82	0.5	2.5	2.7	2.9	1.5	1.7	1.3	2.8	4.9	5
	*Coverage*	95	95.1	96	96	96.7	95	96	97	96.7	97	96	94.5

G	Bias (*H*_0_)	1 × 10^−5^	2 × 10^−5^	−3 × 10^−5^	−5 × 10^−5^	1 × 10^−5^	4 × 10^−5^	2 × 10^−5^	−2 × 10^−5^	5 × 10^−5^	0 × 10^−5^	0 × 10^−5^	3 × 10^−5^
	Bias (*H*_1_)	-6.1	-5.5	-4.9	-8.2	-6.5	-5.5	-5.4	-4.1	-3.5	-7.9	-8.5	-9
	*Coverage*	95.1	94	95	95.3	94.2	95.1	95.5	94.4	95.2	96	95.3	95.1

**Table 4:** Event times (min) for a 10 min treadmill test in a 2 × 2 crossover clinical trial.

Placebo-drug sequence					Drug-placebo sequence				
	Period 1 (placebo)		Period 2 (drug)			Period 1 (drug)		Period 2 (placebo)	
Subject (age)	X_1_	Y_1_	X_2_	Y_2_	Subject (age)	X_1_	Y_1_	X_2_	Y_2_
1 (52.91)	1.5	1	1	1.5	2 (65)	1	1	1	2.5
3 (45.79)	6	4	3.5	>10	4 (40)	6	>10	2.5	2.5
5 (64.37)	1	1	1.5	4.5	6 (59.08)	3	2	1	.5
7 (54.13)	3.5	1.5	.5	3	8 (63.46)	2.5	2.5	1.5	2
9 (61.14)	.5	1	3.5	8	10 (51.78)	2	2.5	2.5	3
11 (47.59)	6	10	6	>10	12 (58.59)	1.5	4.5	2.5	1
13 (70)	.5	.5	1	>10	14 (55.08)	3.5	5.5	4.5	9.5
15 (57.28)	1	1	1	2.5	16 (65.16)	1	2	2	>10
17 (59.75)	1.5	1	.5	.5	18 (41.34)	6	>10	5	3.5
19 (67.77)	1	1.5	2	4	20 (59.44)	2	3	1.5	1.5
21 (42.91)	5	5.5	3	1.5	22 (65.54)	1.5	2.5	1.5	.5
23 (50.72)	2.5	5	6	4.5	24 (70)	1.5	3.5	2.5	3
25 (47.01)	5	5.5	4.5	6	26 (55.63)	3.5	9	6	6
27 (62.26)	1	2	2.5	8.5	28 (62.93)	2	5.5	3.5	8
29 (40)	5	5.5	3.5	2	30 (56.67)	2.5	2.5	1	.5
31 (66.27)	.5	1	2	7.5	32 (63.13)	2.5	3.5	2.5	4
33 (48.16)	5	4	2	2	34 (41.62)	5.5	3	1	.5
35 (65.04)	.5	.5	1	1.5	36 (55.70)	3	5.5	5	.5
37 (66.49)	1.5	2	3	3	38 (66.62)	.5	1	1	5.4
39 (43.19)	6	4	1.5	.5	40 (53.10)	2.5	5	2.5	.5
Median	1.5	1.75	2	3.5	Median	2.5	3.25	2.5	2.5

X_1_: baseline response in period 1; Y_1_: posttreatment response in period 1; X_2_: baseline response in period 2; Y_2_: posttreatment response in period 2.

**Table 5:** Estimated survival time of censored observations in treadmill test for the multiple imputation method, the proposed method, and the proposed method by adding age to the model.

Placebo-drug sequence			Drug-placebo sequence		
Model	Subject (age)	Period 2 (drug)	Subject	Period 1 (drug)	Period 2 (placebo)
		Y_2_		Y_1_	Y_2_
Multiple imputation	3	14.14 ± 0.35	4	14.41 ± 0.39	_
	11	21.46 ± 0.40	16	_	10.63 ± 0.37
	13	10.62 ± 0.41	18	23.34 ± 0.51	_
MERL	3	15 ± 0.30	4	15.1 ± 0.33	_
	11	23 ± 0.38	16	_	11.47 ± 0.32
	13	11.5 ± 0.40	18	24.58 ± 0.48	_
MERL (adding age)	3 (45.79)	16.1 ± 0.28	4 (40)	16.1 ± 0.31	_
	11 (47.58)	24 ± 0.31	16 (65.16)	_	12.5 ± 0.30
	13 (70)	12.2 ± 0.35	18 (41.34)	25.1 ± 0.43	_

Y_1_: posttreatment response in period 1; Y_2_: posttreatment response in period 2.

## Data Availability

The [table] data used to support the findings of this study are included within the article.

## References

[1] Xu R., Mehrotra D. V., Shaw P. A. (2018). Incorporating baseline measurements into the analysis of crossover trials with time-to-event endpoints. *Statistics in Medicine*.

[2] Berry D. A. (2016). *Statistical methodology in the pharmaceutical sciences*.

[3] Chen X., Meng Z., Zhang J. (2012). Handling of baseline measurements in the analysis of crossover trials. *Statistics in Medicine*.

[4] Kenward M. G., Roger J. H. (2010). The use of baseline covariates in crossover studies. *Biostatistics*.

[5] Senn S. S., Senn S. (2002). *Cross-over trials in clinical research*.

[6] Hills M., Armitage P. (1979). The two-period cross-over clinical trial. *British Journal of Clinical Pharmacology*.

[7] Metcalfe C. (2010). The analysis of cross-over trials with baseline measurements. *Statistics in Medicine*.

[8] Yan Z. (2013). The impact of baseline covariates on the efficiency of statistical analyses of crossover designs. *Statistics in Medicine*.

[9] Mehrotra D. V. (2014). A recommended analysis for 2× 2 crossover trials with baseline measurements. *Pharmaceutical Statistics*.

[10] Kimchi A., Lee G., Amsterdam E., Fujii K., Krieg P., Mason D. T. (1983). Increased exercise tolerance after nitroglycerin oral spray: a new and effective therapeutic modality in angina pectoris. *Circulation*.

[11] Markman J. D., Frazer M. E., Rast S. A. (2015). Double-blind, randomized, controlled, crossover trial of pregabalin for neurogenic claudication. *Neurology*.

[12] Grieve A. P. (2016). Crossover versus parallel designs. *Statistical methodology in the pharmaceutical sciences*.

[13] Lewis J. A. (2004). Migraine trials: crossover or parallel group?. *Neuroepidemiology*.

[14] Van Belle G. (2011). *Statistical rules of thumb*.

[15] Brittain E., Follmann D. (2011). A hierarchical rank test for crossover trials with censored data. *Statistics in Medicine*.

[16] Jeong J.-H. (2014). *Statistical inference on residual life*.

[17] Xue-Kun Song P. (2000). Multivariate dispersion models generated from Gaussian copula. *Scandinavian Journal of Statistics*.

[18] Grover G., Gupta V. K. (2014). Multiple imputation of censored survival data in the presence of missing covariates using restricted mean survival time. *Journal of Applied Statistics*.

[19] Liu L. X., Murray S., Tsodikov A. (2011). Multiple imputation based on restricted mean model for censored data. *Statistics in Medicine*.

[20] Sun L., Song X., Zhang Z. (2012). Mean residual life models with time-dependent coefficients under right censoring. *Biometrika*.

[21] Ahmed S. E. (2014). *Penalty, shrinkage and pretest strategies: variable selection and estimation*.

